# Concurrent Urachal Abscess and Urinary Bladder Tumour: Case Report

**DOI:** 10.7759/cureus.31417

**Published:** 2022-11-12

**Authors:** Wassim Alaoui Mhammedi, Mohamed Mokhtari, Anouar El Moudane, Ali Barki

**Affiliations:** 1 Urology, Mohammed VI University Hospital, Mohammed First University, Oujda, MAR; 2 Urology, Centre Hospitalier Universitaire Mohammed VI, Oujda, MAR; 3 Urology, Mohamed VI University Hospital, Oujda, MAR; 4 Urology, Mohammed IV University Medical Center, Oujda, MAR

**Keywords:** case report, malignancy, urothelial carcinoma, bladder neoplasm, abscess, urachus

## Abstract

The urachus is an embryological remnant that normally regresses before birth. Failure in this regression may give rise to different pathologies. Among these, a urachal abscess may be difficult to diagnose based on the clinical presentation alone since this condition can mimic different pathologies. We report the first case of concurrent urachal abscess and invasive low-grade urothelial carcinoma of the urinary bladder.

## Introduction

The urachus originates from the allantois and cloaca during embryogenesis. It normally regresses and obliterates, giving rise to the median umbilical ligament that extends from the dome of the bladder to the umbilicus [1,2]. When the process of regression fails, this can result in many possible pathological conditions, especially a urachal abscess, which occurs as a result of infection and malignant transformation [3,4]. The diagnosis of a urachal abscess can be very challenging and distinction from a malignant pathology can sometimes be impossible based only on clinical and imaging data [5,6]. We report a 75-year-old male who was admitted to our hospital’s urology department for acute abdominal pain and a rapidly growing infraumbilical mass. Ultrasound and a computerized tomography scan identified a urachal abscess with a thickening of the anterior urinary bladder wall and bilateral ureterohydronephrosis. A cystoscopy examination revealed numerous exophytic tumors protruding from the anterior wall. The blood test results were all normal. Pathology confirmed a urachal abscess and revealed a concurrent, invasive, low-grade urothelial carcinoma of the anterior wall of the urinary bladder.

## Case presentation

We report a 75-year-old male who was admitted to our hospital’s urology department for acute abdominal pain and a rapidly growing infraumbilical mass. The patient has no significant medical or surgical history. He reported fever and denied lower urinary tract symptoms or hematuria. Clinical examination at admission revealed a conscious patient. The temperature was 39.4 degrees. Blood pressure, respiratory rate, and pulse per minute were normal. Abdominal and pelvic examination showed an infraumbilical subcutaneous fluctuating mass, measuring 10 cm in diameter. The overlying skin was moderately inflammatory. The abdominal and pelvic ultrasound examination identified a 7-cm heterogeneous pelvic mass with mixed echogenicity on the infraumbilical region located anterior to the urinary bladder. This mass was compatible with an abscess and contained many fistulas. The anterior urinary bladder wall thickened with the identification of many intra-cavitary projections. Bilateral ureterohydronephrosis was also identified with the right kidney pelvis measuring 30 mm in diameter and the left kidney pelvis measuring 35 mm in diameter. An abdominal and pelvic contrast-enhanced computerized tomography scan was performed and confirmed the presence of an enhancing pelvic, infra-umbilical, 12 x 13 x 6 cm mass, with a hypodense central area and a more enhancing peripheral capsule-like area. The mass was closely abutting the bladder dome and abdominal wall with adjacent fat stranding. CT scan also confirmed the presence of diffuse thickening of the urinary bladder anterior wall, with no enlarged pelvic lymph nodes or distant metastases. A cystoscopy examination revealed numerous exophytic tumors protruding from the anterior wall. Complete resection of the observed tumors was performed. Serum biochemistry was normal. Carcinoembryonic antigen was at 15 ng/ml and carbohydrate antigen 125 at 20 units/ml. Urinalysis showed microscopic hematuria.

The patient underwent open surgical urachal abscess drainage. Intraoperative exploration showed that the abscess was surrounding the urachal duct, with the presence of many fistulas. This collection has a capsule and was abutting the anterior urinary bladder wall. The patient received antibiotherapy based on intravenous amoxicillin and clavulanic acid at 1 g per day.

**Figure 1:**
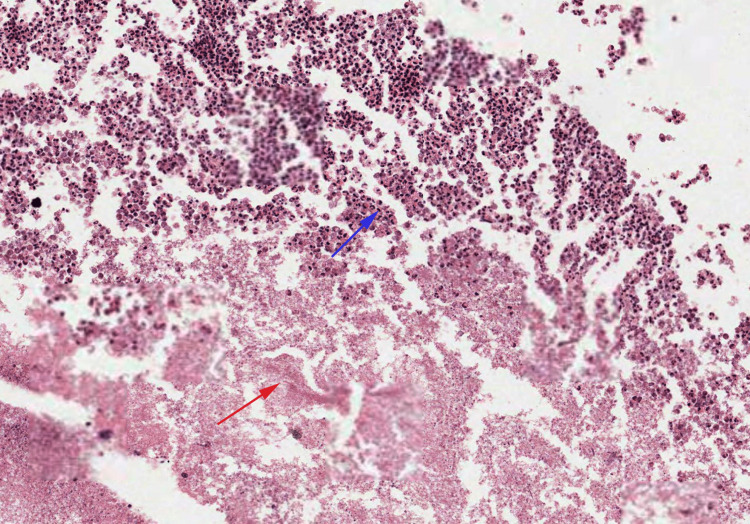
Microphotograph of material from the urachal abscess showing numerous neutrophils (red arrow) with the presence of necrotic material (blue arrow)

Pathology assessment of the drained material revealed abscess and necrotic material (Figure 1).

**Figure 2:**
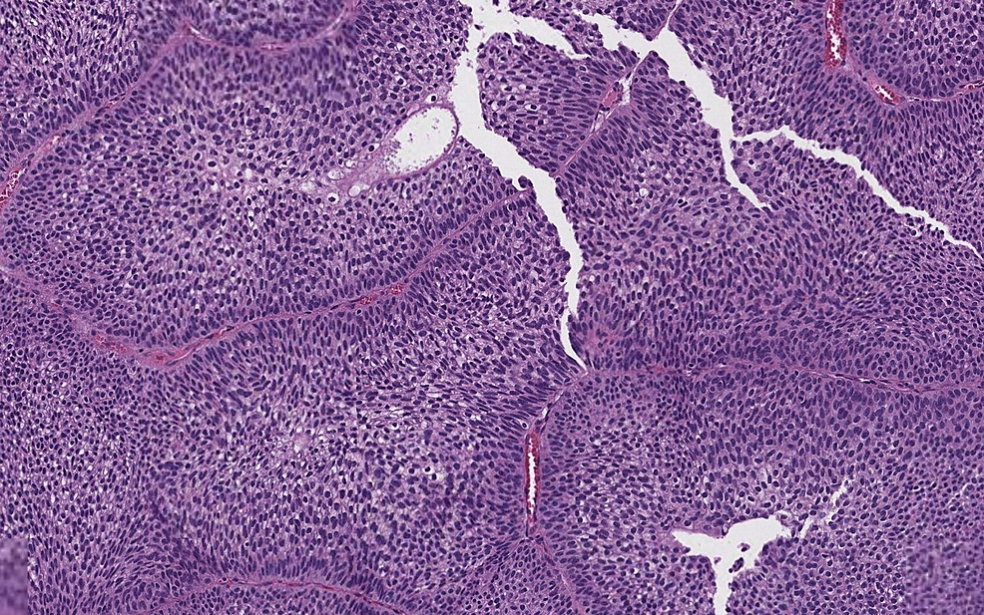
Microphotography showing papillary proliferation layered by neoplastic cells with no nuclear pleomorphism or hyperchromasia. They have a low nuclear/cytoplasmic ratio and mitotic figures are rare.

Pathology assessment of the urinary bladder resection specimen showed a papillary proliferation of carcinomatous cells. These cells are sometimes observed invading the lamina propria, without muscularis propria invasion, in the form of nests or single cells. The neoplastic cells showed no nuclear pleomorphism or hyperchromasia. They have a low nuclear/cytoplasmic ratio and mitotic figures are rare (Figure 2). The diagnosis of concurrent urachal abscess and invasive pT1 low-grade urothelial carcinoma of the urinary bladder was established. The decision was to follow up with the patient and to conduct local intravesical Bacillus Calmette-Guerin therapy.

## Discussion

Microbial dissemination to the urachus can be achieved by many means, especially lymphatic and hematogenous, or from the adjacent urinary bladder [7]. This would result in urachal remnants infection, which classically affects young adults and children [7]. A wide spectrum of gram-positive and gram-negative organisms can be responsible for such infections [8]. In severe infection cases, urachal abscess formation can occur. The insidious nature of the clinical presentation in cases of urachus remnants infection can explain the sufficient duration for abscess formation [7].

The major differential diagnosis in urachal pathology remains to be malignant urachal neoplasms. These represent less than 0.5% of all bladder malignancies, with adenocarcinoma being the most frequently described histological type. While a malignant transformation of the urachus occurs in middle or old-aged patients, infection and possible abscess formation classically occur in children and young adults [7]. The location for urachal carcinoma is the juxta-vesical area of the urachus. This would explain possible extension to the umbilicus and to the bladder wall in the late stages [9].

At the clinical level, hematuria is the most frequent symptom and is reported in 70% of cases of urachal abscesses. Abdominal mass, lower urinary tract symptoms, abdominal pain, and discharge from the umbilicus are other reported symptoms [3].

Our patient had symptoms essentially related to the urachal abscess, whereas he reported no hematuria and no urinary tract symptoms. Imaging and later pathological confirmation enabled confirmation of the concurrent bladder neoplasm.

At the radiological level, ultrasonography shows a urachal abscess as a midline, heterogeneous mass in continuity with the bladder. CT scan shows a urachal abscess, as well as urachal malignancies as a heterogeneously enhanced mass containing solid, cystic, or solido-cystic configuration. Both cases may present areas of low density, related to an abscess in cases of urachal abscess and to mucinous areas in cases of urachal adenocarcinoma [3,9].

Imaging can help distinguish the malignant disease from the urachal abscess by identifying features of malignancy such as the presence of calcifications (observed in 50-70% of malignancies) and bladder wall involvement [9].

On the therapeutic level, in adults, a urachal abscess usually is based on broad-spectrum antibiotics and drainage. Reinfection can occur in 30% of cases after initial treatment [7].

## Conclusions

A urachal abscess is a rare condition in adults. Distinguishing a urachal abscess from malignancy is challenging based on clinical presentation and imaging studies. In our case, extensive explorations enabled the identification of a concurrent bladder tumor. This is the first reported case of concurrence between these two entities. Our work proves that establishing a diagnosis of urachal abscess doesn’t exclude association with other diseases.

## References

[1] Ashley RA, Inman BA, Routh JC, Rohlinger AL, Husmann DA, Kramer SA (2007). Urachal anomalies: a longitudinal study of urachal remnants in children and adults. J Urol.

[2] Walker C (2010). A case report of urachal abscess: a rare differential in adult abdominal pain. Hawaii Med J.

[3] Parada Villavicencio C, Adam SZ, Nikolaidis P, Yaghmai V, Miller FH (2016). Imaging of the urachus: anomalies, complications, and mimics. Radiographics.

[4] Chen YH, Tseng JS (2022). Concurrent urachal abscess and florid cystitis glandularis masquerading as malignancy: a case report and literature review. BMC Surg.

[5] Ninitas P, Anselmo MP, Silva AC, Ferreira AI, Santos JF (2019). Urachal abscess mimicking malignant tumor: can imaging tell them apart?. Acta Radiol Open.

[6] Lam SW, Linsen PV, Elgersma OE (2019). Infection of previously closed urachus mimicking malignancy: a case report and literature review of radiological fndings to the diagnosis. Clin Med Insights Case Rep.

[7] Smith AK, Hansel DE, Jones JS (2008). Role of cystitis cystica et glandularis and intestinal metaplasia in development of bladder carcinoma. Urology.

[8] Elkbuli A, Kinslow K, Ehrhardt JD Jr, Hai S, McKenney M, Boneva D (2019). Surgical management for an infected urachal cyst in an adult: case report and literature review. Int J Surg Case Rep.

[9] Yu JS, Kim KW, Lee HJ, Lee YJ, Yoon CS, Kim MJ (2001). Urachal remnant diseases: spectrum of CT and US findings. Radiographics.

